# Response surface methodology based optimization and scale-up production of amylase from a novel bacterial strain, *Bacillus aryabhattai* KIIT BE-1

**DOI:** 10.1016/j.btre.2020.e00506

**Published:** 2020-07-15

**Authors:** Sanjay Kumar Ojha, Puneet Kumar Singh, Snehasish Mishra, Ritesh Pattnaik, Shubha Dixit, Suresh K. Verma

**Affiliations:** a School of Biotechnology, Kalinga Institute of Industrial Technology (KIIT) Deemed-to-be-University, Bhubaneswar, 751 024, India; b Pandorum Technologies Pvt. Ltd., Bangalore Bioinnovation Centre, Helix Biotech Park, Electronic City Phase 1, Bengaluru, 560 100, India; c School of Pharmacy, Lloyd Institute of Management and Technology, PlotNo.11, Knowledge Park II, Greater Noida, 201310, India

**Keywords:** Amylase, *Bacillus*, Batch fermentation, GenBank, KIIT BE-1, Response surface methodology

## Abstract

A novel strain KIIT BE-1 isolated from a specialized environment, screened through starch iodine test from a set of eighty-five biodigestate isolates, produced amylase maximally when cultured for 48 h at 37 °C. The molecular and biochemical characterization confirmed it as a strain of *Bacillus aryabhattai*. It exhibited optimal amylase activity (3.20 U/ml) at 36 h post incubation with a media combination of starch and yeast extract for C-N source respectively. Statistical optimisation by response surface modeling showed R^2^ values of 0.9645 for biomass and 0.9831 for amylase activity, suggesting the significance of the model. The optimised medium (10.25 % starch, 5.0 % peptone, 5.18 % yeast extract, pH 7.3) enhanced the enzyme activity to 4.16 U/ml (1.39-fold) from 3.20 U/ml of un-optimised medium. Further, the biomass yield and the enzymatic activity in optimized medium and process conditions increased by 1.14 and 1.21 folds subjected to a 5 l scaled-up operation in a lab-scale bioreactor.

## Highlights

• *Bacillus aryabhattai* KIIT BE-1 (GenBank,NCBI, Access. No. KC610087) was identified.
• Starch & yeast extract as carbon and nitrogen sources showed best amylase production.
• A 1.39 fold enhanced amylase activity was observed during media optimization.
• The enzymatic activity demonstrated a 1.21 fold increase in bioreactor.
• The biomass yield was increased 1.14 fold in bioreactor.

## 1 Introduction

The genus *Bacillus* represents a well-diversified group of microbes of human economic significance. The protease of the facultatively anaerobic, reportedly the biggest (length 4 μm, dia. 1.5 μm) known bacterium, *Bacillus megaterium* [1] found in diverse habitats, is widely used in detergent industry [2]. Another soil *Bacillus* spp., *B. licheniformis*, produces alkali-tolerant protease suitable for the laundry industry [3] and lipase [4] for food industry. *Bacillus pumilus* from soil produces xylanase [5] which suitable for pulp and paper industry. Biopesticide from *B. subtilis* QST 713 potentially combats infectious fungi by various mechanisms including direct competition for nutrients (competitive exclusion) [6].

Different strains like *Bacillus subtilis*, *Bacillus stearothermophilus*, *Bacillus licheniformis* and *Bacillus amyloliquefaciens* are efficient producers of *α*-amylase for various applications [7]. *Bacillus* is a suitable candidate for commercial enzymes production as it maintains its native folded state, *i.e.*, natural unaltered state, against the various harsh steps in bioprocess engineering, and also it secretes proteins directly to the extracellular medium facilitating its easy extraction and purification thereby saves process time, cost and complications.

Although amylases are found in all life forms, microbial *α*-amylases are popular in industries [8]. *Aspergillus* [9], *Streptomyces* [10], and *Bacilli* [11] (*viz*., *B. subtilis* [12], *B. stearothermophilus* [13], *B. licheniformis* [14], *B. amyloliquefaciens* [15]) are good commercial *α*-amylase producers with varying (thermostability, acid-tolerance *etc.*) properties. Importantly, microbial amylases have entirely replaced chemical processes in industrial starch processing [16]. While large-scale microbial *α*-amylase production can occur with glucose and maltose, starch is the preferred substrate for economic reasons. *Aspergillus oryzae* [17], *Streptomyces* sp. [18], *Halomonas meridiana* [19] and *Bacillus* sp. IMD 435 [20] produced *α*-amylase with yeast extract as the nitrogen source.

As the composition of media strongly influence growth and enzyme production, optimising the media components and the cultural parameters are essential in standardising a bioprocess [21], and therefore the statistical methods such as the response surface methodology (RSM) are useful [22]. Pedersen and Nielsen [17] reported a 110–156 % increase in the *α*-amylase production by *A. oryzae* with yeast extract nitrogen supplementation compared to using ammonia alone.

The limitations of the single factor optimisation are eliminated by employing RSM to explain the combined effects of various critical factors [23]. RSM is a collection of experimental strategies, mathematical methods and statistical inference for constructing and exploring an approximate functional relationship between response variable and a set of design variables. It determines the interactive effect of variables on the responses by quantifying the relationship between the controllable input parameters and the obtained response surfaces, and reduces the number of wet-lab experiments without upsetting the accuracy of the results. A full-factorial central composite design is usually used to acquire data to fit an empirical second-order polynomial model [24]. With this background, a study was designed to isolate, identify and characterise a few biomethanation microbes, and to optimise the media and the process for maximal enzyme activity. A scaled-up operation ensued to validate the medium and process optimisations.

## 2 Results and discussion

### 2.1 Microbiological and basic biochemical analyses

Amylase production process optimisation being the major objective, the isolate KIIT-BE1 showing the maximal amylase activity during the plate assay (Fig. 1A) was selected. The 48 h nutrient agar colonies exhibited irregular shape with whitish creamy appearance. Gram staining followed by microscopic observation (40X Nikon) revealed that the isolate was Gram positive rod (Fig. 1B). The photomicrograph of the isolate is presented in Fig. 2 which further confirms the structural characteristic as rods.

Biochemical analyses (Table 4) showed that the isolate was positive to catalase, Voges-Proskauer, amylase, gelatinase, lecithinase, chitinase and pectinase, whereas negative to tributryn hydrolysis, casein hydrolysis, urease and citrate utilisation.

### 2.2 Molecular phylogeny analyses

The isolate identification at the molecular level was performed using the EzTaxon server (www.ezbiocloud) [25] based on the 16S rRNA sequence fragments. The BLAST followed by the sequence alignment confirmed that KIIT BE-1 was a *Bacillus* (Access. No. KC610087.1), with a maximal similarity with *Bacillus aryabhattai* B8W22(T) (Access. No. EF114313) [26], *Bacillus megaterium* IAM 13418(T) (D16273) [27], *Bacillus flexus* IFO 15715(T) (AB021185) [28], *Bacillus paraflexus* RC2(T) (FN999943) [29] and *Bacillus qingshengii* G19(T) (JX293295) [30] with 99.64, 99.64, 99.0, 97.91 and 97.86 % pair-wise similarity, respectively (Fig. 3).

The bases number (similarity calculation achieved without considering gaps) compared in EzTaxon server was 10 and the number of matched bases was 9 (*i.e.*, 90 % similarity; a conservative measure). The similarity values will be lower in most cases containing higher sequencing errors if the gaps were considered. In the molecular phylogeny construct, this remains as a limitation.

### 2.3 Enzyme production vis-à-vis physical parameters

Amylase activity trend of KIIT BE-1 in the starch-supplemented production medium showed that the maximal amylase activity (3.2U/ml) was at 36 h, steadily decreasing thereafter (Fig. 4). Comparison of the bacterial population with the amylase activity showed that the activity was proportionate with the biomass growth, demonstrating that active amylase was growth-associated [31].

Temperature-wise, the highest amylase production that was observed was at 37 °C (Fig. 5). The percent amylase production increased when the agitation increased to 150 rpm (Fig. 6), and this increased production efficiency could be attributed to the better mass transfer, thereby supplementing to growth. Beyond 150 rpm, the production efficiency was speed neutral. Thus, for further studies including that of the scaled-up bioreactor, the optimal physical conditions provided were, temperature 37 °C, agitation 150 rpm, and a neutral pH, for 36 h duration.

### 2.4 Starch as the carbon source

The various carbon sources (starch, galactose, glucose, fructose and sucrose), provided to KIIT BE-1, maximum bacterial growth was observed in starch (Fig. 7) attributable to the fact that starch is one of the mostly abundant polysaccharides, contributed in natural microbial amylase production [32]. The activity decreased by 40 % with the availability of short-chain carbohydrates such as the monosaccharides. Addition of glucose as a carbon source suppressed the expression of amylase gene in *Sulfolobus solfataricus* [32] and *Bacillus* [33]. Galactose was found to be the next most efficient carbon source for amylase activity after starch with KIIT BE-1, whereas glucose, fructose and sucrose inhibited amylase production. Roy et al. [33] have also discussed about glucose as catabolic suppressor for carbohydrate metabolic enzymes.

### 2.5 Yeast extract as the nitrogen source

The amylase activity of KIIT BE-1 was maximal with yeast extract compared to various other organic and inorganic nitrogen sources (Fig. 5). Though the organic sources such as tryptone, beef extract and peptone showed a similar trend, inorganic nitrogen sources affected the production negatively. Gupta et al. [34] also reported a similar observation.

Hashemi et al. [35] reported better amylase production with inorganic nitrogen sources by some bacterial species, possibly due to ecological reasons. For instance, microbes from the arid regions reportedly prefer inorganic nitrogen sources for amylase production. Ammonium sulphate, which did not show any amylase production effect on KIIT BE-1, was found to be efficiently enhancing amylase production in different *Aspergillus* sp. [34]

### 2.6 Statistical analyses and model validation

Experiments were performed in triplicates for the sets of combinations generated from design matrix and a total of 30 sets were generated (Table 3). These results were fitted to the third order model equation by applying multiple regression analysis for biomass yield and amylase activity, and a second order polynomial equation represented the experimental data (Eqs. (2) and (3)). The responses for the given levels of each factor were predicted based on these equations in terms of coded factors. By default, the high and the low levels of the factors were coded as +1 and -1 respectively. Through comparison of the factor coefficients, the equation facilitated the identification of the relative impact of the factors.

(2) Biomass yield = + 0.68 + 0.054*A - 2.083E-003*B + 9.583E-003*C + 7.917E-003*D + 0.013*AB + 8.125E-003*AC - 6.250E-004*AD - 0.017*BC - 6.250E-004*BD - 0.013*CD 0.068*A^2^ - 6.563E-003*B^2^ - 0.044*C^2^ - 0.11*D^2^

(3) Amylase activity = + 3.86 - 0.14*A - 0.12*B - 0.059*C + 0.11*D - 0.085*AB + 0.021*AC - 0.099*AD - 0.15*BC - 0.23*BD - 0.20*CD - 0.29* A^2^ + 0.011*B^2^ - 0.56*C^2^ - 0.60*D^2^

The F values for biomass yield and amylase activity are 6.93 and 9.06 respectively, implying that the models were significant (Table 4). High coefficient of determination (R^2^) value indicated the capability of third-degree equation in representing the system under the given experimental domain. The R^2^ values were 0.9645 for biomass and 0.9831 for enzyme activity, suggesting that the model could explain 96.45 and 98.31 % of total variation in the biomass yield and amylase activity, respectively.

The adequate precision value (measurement of the signal to the noise ratio) of 8.930 for biomass yield and 10.061 for amylase activity indicated an adequate signal (a value greater than 4.0 is desirable) suggesting a negative design space using this model [36]. The p-values for each coefficient (indicating high significance of corresponding coefficient) are listed in Table 4. It is obvious from the data that squared term of pH (D^2^) is variable with the largest effect. It suggests, although *Bacillus* reportedly withstands various stress conditions, KIIT BE-1 is quite pH sensitive for biomass yield and amylase activity. Next higher coefficient (A^2^) is starch, supporting earlier observation [37]. To see the interaction of different coefficients for maximal biomass yield and amylase activity by KIIT BE-1, the graphs (Fig. 7) were plotted by Design Expert v9.1. A combined effect of the yeast extract and starch had a maximum positive influence on the biomass yield and the amylase activity (Figs. 7A, D).

### 2.7 Validation of the experimental data

Different variables fairly and differentially influenced biomass yield and amylase production. Fig. 10 showing the points of the observed *vs* the predicted results for biomass production (Fig. 6A) and enzyme activity (Fig. 6B) confirmed this assumption. The amylase activity was maximal with starch and yeast extract in the ranges between 9.50 and 10.50, and 4.70 and 5.30 g/l, respectively. Gangadharan et al. [38] reported a similar combined effect. The pH conjointly had a similar effect on the biomass yield and amylase activity, the optimal range being 6.5–7.5. This observation supports earlier report of Gupta et al. [34] who reported a pH range of 6–7 to produce amylase from *Bacillus*.

The optimised predicted values for maximal biomass production (0.82) and enzyme activity (4.16 U/ml) are, starch 10.25 %, peptone 5.0 %, yeast extract 5.18 %; pH 7.3. The realised values for the biomass yield and the amylase activity were 0.73 and 4.08 U/ml, appreciably close to predicted values, thus validating the model. Roy et al. [33] opined the need of multiple nitrogen sources for amylase production. The data were validated through confirmatory experiments performed in triplicates. A 1.39-fold increase in amylase activity against unoptimised medium was achieved in the present study authenticating the efficacy of RSM in process optimisation. Gangadharan et al. [38] reported a high substrate affinity of amylase towards soluble starch and that the substrate concentration influenced the amylase activity.

### 2.8 Scaled-up study

Once the parameters were standardised in the shake-flasks culture, the experiment was scaled-up to a process-controlled bioreactor. Assumedly, the enzyme production in a bioreactor is higher than in shake-flasks culture as the various critical variable factors such as the dissolved oxygen (DO) and the pH can be optimally controlled at the desired levels [33]. During the study, a regular monitoring of various parameters were done to confirm the deviations if any, maintaining the pH between 6.8 and 7.7. A sharp reduction in DO observed at initial level (growth phase) was maintained at 80 % throughout. The initial reduction could be due to the oxygen consumed by the actively growing microbes, and it remained constant when stationary phase set in. The biomass yield increased 1.14-fold, and the enzymatic activity 1.21-fold. In similar studies using bioreactor, Elmarzugi et al. [37] reported a 1.14-fold and Enshasy [38,39,40] achieved 1.63-fold increments in amylase activity. Sharma and Satyanarayana [11] achieved an overall 2.4-fold increase in amylase activity in batch fermentation under laboratory conditions by *Bacillus acidicola*. Amylase is one of the very useful enzymes for the hydrolysis of starchy substrate thus it can be utilised as pre-hydrolytic enzyme before the anaerobic digestion of starchy waste such as kitchen refuse [41–48].

## 3 Materials and methods

### 3.1 Microbial isolation and identification

A total of 85 bacterial strains from the biodigester digestate were isolated and maintained at 37 °C on nutrient agar (Hi Media, India) slants (agar 1.8 %; pH 7.0), and their starch-degrading abilities were confirmed through starch-iodine plate screening. The isolates were cultured (37 °C; 100 rpm) in a shaker incubator (Innova New Brunswick, Germany) in nutrient broth, and preserved at −80 °C in 60 % (v/v) glycerol. Isolates were further scanned by electron microscope (Merlin, Zeiss) to observe its physical and structural uniqueness.

For plate assay, the bacterial colonies were incubated (37 °C, 48 h) on modified nutrient agar (1.00 % soluble starch, 0.40 % yeast extract, 1.00 % peptone, 0.05 % MgSO_4_, 0.05 % NaCl, 0.02 % CaCl_2_, 2.00 % agar; pH 7.0) plates. Their starch degradation was confirmed through starch-iodine plate screening wherein the amylase producing (positive) colonies exhibited clear zones around upon flooding with iodine solution. These amylase positive isolates were biochemically characterised as per the Bergey’s Manual of Systematic Bacteriology [49].

### 3.2 Microbial identification - 16S RiboProfiling

The DNA from the culture gave a single high-molecular weight band on 1.2 % agarose gel when evaluated for the quality. Upon agarose gel resolution, the PCR-amplified 16S rDNA gene fragment showed a single discrete 1500bp PCR amplicon band. The purified PCR amplicon was subjected to forward and reverse DNA sequencing with 8 F and 1492R primers using BDT v3.1 Cycle Sequencing Kit on ABI 3730xl Genetic Analyser. From the forward and reverse sequence data, the consensus sequence of 1403bp 16S rDNA gene was generated using Aligner Software.

For the sequence and phylogenetic analyses, the 1403bp 16S rDNA was subjected to BLAST with the online NCBI database. Based on the maximum identity score, the first 22 sequences were selected and aligned using multiple alignment software program Clustal W. Neighbor-joining method [50] was used to infer the evolutionary history, and optimise the tree (drawn to scale) with the sum (0.52653124) of the branch length. The bootstrap test (with 1000 replicates) percentages of replicate trees where the associated taxa clustered together are shown next to the branches [51]. The branch lengths are in the same units as those of the evolutionary distances (ED) to infer the phylogenetic tree. Kimura 2-parameter method [52] was used to compute the EDs in the units of the number of base substitutions/sites. Evolutionary analyses were conducted using MEGA6 software [53].

### 3.3 Inoculum preparation

A loop-full of the viable cells was grown (37 °C, 24 h, 150 rpm) initially in nutrient broth, and then in fresh culture medium. About 60 ml of the medium with (2 % v/v) active cells was centrifuged at 6000 rpm for 10 min containing 4 × 10^8^ CFU/ml. The centrifuged pellet was washed with sterile normal saline (0.85 % NaCl) solution several times to obtain a final 0.8–0.9-unit absorbance at 600 nm [53].

### 3.4 Biomass determination

The biomass was determined as per protocol described by Singh et al. [54], with modifications. The fermented broth sample was diluted five times and the biomass was estimated sequentially at 600 nm, and 620 nm respectively. The cell free medium was used as blank.

### 3.5 Enzyme production

The amylase production was carried out in modified nutrient broth medium as mentioned earlier (10.0 soluble starch, 10.0 peptone, 0.5 NaCl, 0.5 MgSO_4_.7H_2_O, 0.2 CaCl_2_, and 4.0 yeast extract, all values in g/l; pH 7.0). 50 ml of the culture medium in a 250 ml capacity Erlenmeyer flask was inoculated with 0.5 ml of the overnight culture and incubated at 37 °C in a shaker-incubator (Innova New Brunswick, Germany; 150 rpm; 144 h). The samples were harvested in triplicates and the cells separated (10,000 rpm; 15 min) at 4 °C in a refrigerated centrifuge (Sigma-Japan). The enzyme in the supernatant was assessed and characterized.

### 3.6 Effect of critical physical parameters on enzyme production

To optimise the incubation period, samples were drawn from the isolate cultured in the modified nutrient broth medium at 6 h intervals and the residual activities determined under standard assay conditions. Studies suggests that the two most critical parameters affecting the amylase production besides incubation period are agitation and temperature. The temperature range for *Bacillus* is reportedly 30–40 °C [55–60], and up to 50 °C in thermophiles [50]. Similarly, the optimal agitation rate is invariably reported as 200 rpm [58–61]. Thus, to corroborate the optimal temperature and agitation regime for the biomass accumulation and active enzyme production by KIIT BE-1, the studies were carried out by varying the temperature from 30 to 55 °C, and the agitation rate from 60 to 210 rpm while keeping all other physicochemical parameters constant.

### 3.7 Effect of nutritional sources on enzyme production

With a major focus on maximizing the enzyme yields from KIIT BE-1, the carbon and nitrogen sources in the production medium were optimised at neutral pH (7.0) while maintaining the temperature and agitation constant at 37 °C and 150 rpm. The effect of the various carbon sources was studied by replacing soluble starch (1 % w/v) with various sugars, such as, cellulose, fructose, galactose, glucose, glycine, lactose, sucrose, starch, xylose, and sodium acetate.

The effect of the nitrogen sources (0.4 % w/v final concentration) was studied by replacing the peptone and yeast extract with various organic (*viz*., malt and beef extracts) and inorganic nitrogen (ammonium sulphate) sources.

### 3.8 Enzyme assay

The enzyme activity was determined (dinitrosalicylate method; estimating the reducing sugar at 545 nm) by hydrolysing starch as the substrate (1 % w/v in 100 mM phosphate buffer; pH 7.0) at 50 °C. The *α*-amylase activity unit (U) is the amount of enzyme capable to produce 1 μmol of glucose/min. Before statistical data analyses and statistically-derived 3D graphs, optimal values of each specific parameter for the maximal biomass yield and enzyme activity were worked out.

### 3.9 Statistical analysis and modeling

Statistical analyses of the model were performed to evaluate the analysis of variance using Design-Expert 9.1 (Stat-Ease, Minneapolis, MN, USA). The full-factorial central composite design consisted of a complete 2 k factorial design, where k is the number of test variables; n_0_ centre points (n_0_≥1) and two axial points on the axis of each design variable at a distance of α ( = 2^k/4^, = 2 for k = 4) from the design centre [24, Table 1]. Hence, the total number of design points was N = 2^k^ + 2k + n_0_. CCRD (central composite experimental design) was used to optimise the biomass and enzyme activity using starch, peptone, yeast extract and pH as the chosen variables (Table 2), and generating a set of 30 experiments with different combinations (Table 3). The biomass yield and the amylase activities were performed in triplicates, and the responses obtained were termed respectively as X and Y (Table 5).

Second order polynomial equation as below was used for ANOVA of the test data obtained through RSM.

(1) Y = β_0_ + β_1_A+β_2_B+β_3_C+β_4_D+β_11_A^2^+β_22_B^2^ + β_33_C^2^ + β_44_D^2^ + β_1_β_2_AB + β_1_β_3_AC + β_1_β_4_AD + β_2_β_3_BC + β_2_β_4_BD + β_3_β_4_CD

where, Y is the predicted response; β_0_ is intercept; β_1_, β_2_, β_3_ and β_4_ are linear coefficients; β_1,1_, β_2,2,_ β_3,3_, β_4,4_ are squared coefficients; β_1,2_, β_1,3_, β_1,4_, β_2,3_, β_2,4_, β_3,4_ are interaction coefficients; and A, B, C, D, A^2^, B^2^, C^2^, D^2^, AB, AC, AD, BC, BD and CD are independent variables.

As per the ANOVA, the effect and regression coefficients of the individual linear, quadratic and interaction terms were determined. The statistical significance of the model equation and the model terms was determined by Fisher’s test. The quality of fit of the second-order polynomial model was expressed *via* the coefficient of determination (R^2^). The fitted polynomial equation expressed as 3D surface plots provided a better visualisation of the relationship between the response and experimental levels of each factor and to deduce the optimal conditions. Point optimisation method was employed to optimise the level of each variable for the maximum response. The combination of the various optimised variables yielding maximal response was determined, and the model was validated with repeated tests [59].

### 3.10 Data validation

Experimental studies with different variables fairly and differentially influencing the biomass yield and amylase production by KIIT BE-1 as reflected through the RSM modeling were carried out to confirm the veracity of the predicted values. The data were validated through confirmatory experiments in triplicates. The deviations between the predicted and actuated values were further analysed.

### 3.11 Scaled-up study

Scaled-up batch production was carried-out in a 7.5 l bench-top fermenter (BioFlo/Celligen 115, New Brunswick, USA) with a 3.0 l working volume. The fermenter was equipped with all the basic automation requirements (pH-mV controller - Mettler Toledo, USA; DO probe - Mettler Toledo, USA; temperature; aeration; and agitation). Other critical (physical) factors like agitation, temperature and aeration were maintained at 100 rpm, 37 °C and 1.0 vvm, respectively. The culture medium was the optimised basal medium having 10.25 % starch, 5.0 % peptone and 5.18 % yeast extract, and pH 7.3. A 1.21-fold increase in amylase activity observed in a scaled-up study,fermenter compared to the batch fermenter in laboratory shaker hints at the possibility of large-scale production of amylase for several industrial applications.

## 4 Conclusion

Amylases comprise 30 % of global enzyme need and used in food, health, paper, detergent and many more industries. With an attempt to study the culturable microbial consortia inside a kitchen-refuse propelled digester looking for microbes with potent amylase activity, a total of 85 isolates from the digester were screened. The best amylase producer was isolated and was identified as *Bacillus aryabhattai* KIIT BE-1 (NCBI GenBank Access. No. KC610087.1). RSM played a key role in media optimisation and a 1.39-fold increment in amylase activity was observed at the end. This statistical software is very useful in scientific studies involving identification of the key factors or the responses of these key factors on the multi-factor dependent variables [15,60,61]. A 1.21-fold increase in amylase activity observed in a scaled-up fermentation hints at the possibility of commercial production of KIIT BE-1 amylase for multiple industrial applications when compared to the flask culture study. Further process validation for the scaled-up production strategies is suggested as a further study to confirm the techno-feasibility of its mass commercial production.

## Ethical statement

This article does not contain any studies with human participants or animals performed by any of the authors.

## Author contributions

The corresponding authors and Snehasish mishra designed the experiments related to potimisation of bacterial amylase production and it’s scale up. They were also involved in writing and editing the manuscript. Sanjay kumar ojha was involved as the key contributor in all the experimental analysis and manuscript preparation. Puneet kumar singh played important role in the analysis of all the data, preparation and improvisation of the manuscript. All authors reviewed and discussed in the manuscript.

## Data availability

The data and the protocols used in the manuscript are available with the corresponding author.

## CRediT authorship contribution statement

**Sanjay Kumar Ojha:** Conceptualization, Methodology, Software, Data curation, Writing - original draft, Funding acquisition. **Puneet Kumar Singh:** Methodology, Data curation, Writing - original draft. **Snehasish Mishra:** Visualization, Investigation, Project administration. **Ritesh Pattnaik:** Data curation, Visualization, Investigation. **Shubha Dixit:** Methodology, Software, Validation, Formal analysis. **Suresh K. Verma:** Conceptualization, Methodology, Investigation, Validation, Writing - original draft, Writing - review & editing, Funding acquisition.

## Declaration of Competing Interest

The authors declare no competing interests.
